# Growth temperature and genotype both play important roles in sorghum grain phenolic composition

**DOI:** 10.1038/srep21835

**Published:** 2016-02-24

**Authors:** Gangcheng Wu, Stuart K. Johnson, Janet F. Bornman, Sarita J. Bennett, Michael W. Clarke, Vijaya Singh, Zhongxiang Fang

**Affiliations:** 1 Food Science and Technology Program, School of Public Health, Curtin University, Perth, WA 6845, Australia; 2 International Institute of Agri-Food Security (IIAFS), Curtin University, PO Box U1987, Perth, WA 6845, Australia; 3 Department of Environment and Agriculture, School of Science, Curtin University, Perth, WA 6845, Australia; 4 Centre for Microscopy, Characterisation and Analysis, University of Western Australia, Perth, WA, 6009, Australia; 5 Queensland Alliance for Agriculture and Food Innovation, The University of Queensland, Brisbane, Qld 4072, Australia; 6 Faculty of Veterinary and Agricultural Sciences, The University of Melbourne, Parkville, Victoria 3010, Australia

## Abstract

Polyphenols in sorghum grains are a source of dietary antioxidants. Polyphenols in six diverse sorghum genotypes grown under two day/night temperature regimes of optimal temperature (OT, 32/21 °C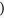 and high temperature (HT, 38/21 °C) were investigated. A total of 23 phenolic compounds were positively or tentatively identified by HPLC-DAD-ESIMS. Compared with other pigmented types, the phenolic profile of white sorghum PI563516 was simpler, since fewer polyphenols were detected. Brown sorghum IS 8525 had the highest levels of caffeic and ferulic acid, but apigenin and luteolin were not detected. Free luteolinidin and apigeninidin levels were lower under HT than OT across all genotypes (*p* ≤ 0.05), suggesting HT could have inhibited 3-deoxyanthocyanidins formation. These results provide new information on the effects of HT on specific polyphenols in various Australian sorghum genotypes, which might be used as a guide to grow high antioxidant sorghum grains under projected high temperature in the future.

Sorghum (*Sorghum bicolor* (L.) Moench) is an important global cereal crop due to its tolerance to high temperatures and arid conditions. The total global sorghum production was around 62.3 million tonnes in 2013 and more than 50% of that was produced in the African and Asian regions^1^. In these two regions, sorghum grains are used as food, such as flat breads, or porridges^2^. However, in developed countries such as the USA and Australia, sorghum grain is primarily used for animal feed and ethanol production^3^.

It has been reported that some sorghum genotypes have high levels of phenolic compounds and strong antioxidant activities^4^. The consumption of foods with high levels of phenolic compounds may assist in prevention of certain age-related diseases^5^,^6^. Results of epidemiological studies suggested that dietary intake of phenolic abundant foods may reduce the incidence of cardiovascular disease, coronary heart disease and a variety of cancers^7^,^8^. In addition, the polyphenols, such as 3-deoxyanthocyanidins, specific to sorghum, have reportedly good anti-inflammatory properties^2^. The level of phenolic compounds in sorghum grains vary widely with genotype as previously reported^9^,^10^. However, most studies only evaluated the polyphenols as a collective group, and information on the differences in specific polyphenols among sorghum genotypes is limited. Though some specific polyphenols in several sorghum genotypes were HPLC analyzed^11^, others without authentic standards might not be identified by HPLC without mass spectrometry.

The Intergovernmental Panel on Climate Change (IPCC) Fourth Assessment have predicted that the global temperature will increase by 1.8 to 4.0 °C by 2100^12^. Understanding variability of polyphenol composition under increased temperature is helpful because the concentration and profile of polyphenols can affect the nutritional values of sorghum grain. Published studies investigating how increased temperature influences the phenolic compounds in plants, have mainly focused on fruits and vegetables. Wang and Zheng^13^ reported that the total phenolic compounds in strawberry increased under high temperature treatments, whereas higher temperatures significantly decreased total phenolic contents in sweet potato leaves^14^. However, no information is available on the effects of elevated temperatures on phenolic compounds in cereal grains, including sorghum.

Therefore, the purpose of this research was to investigate the effects of two different temperature regimes during plant production on sorghum grain phenolic compounds, analysed using high performance liquid chromatography-diode array detection-electrospray ionization mass spectrometry (HPLC-DAD-ESIMS) in six genotypes. The potential for matching sorghum grains with specific phenolic profiles when grown under high temperature conditions to specific food use are discussed.

## Results

### Identification of phenolic compounds

**Figure 1:**
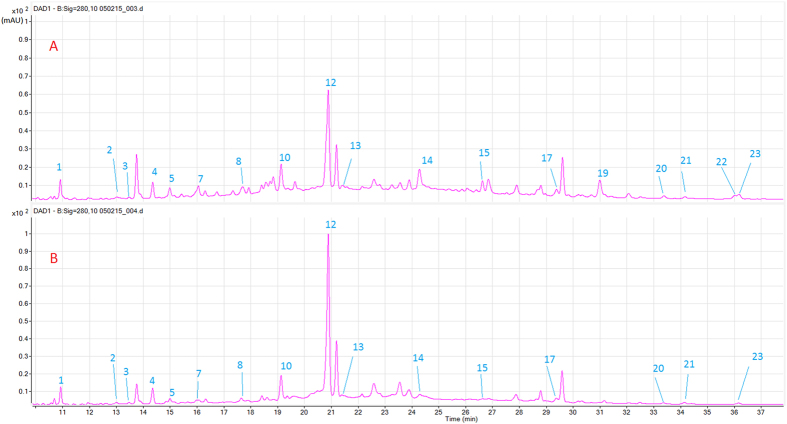
Representative chromatograms of sorghum whole grain phenolic compounds (**A**) Free phenolic compounds from genotype CHH2 grown at OT. (**B**) Free phenolic compounds from CHH2 grown at HT.

The HPLC retention time (Rt), UV absorption maxima and parent and fragment ions of 23 different phenolic compounds positively or tentatively identified in the samples are listed in Table 1. Representative chromatograms of the free polyphenolics in the red-pericarp genotype CHH2 (Fig. 1) are presented. To assist the interpretation of MS fragmentation patterns, the 23 compounds were classified into five groups according to their chemical structures.

Phenolic acids. Phenolic acids consist of hydroxybenzoic acids and hydroxycinnamic acids. Only peak 3 was tentatively identified as a derivative of hydroxybenzoic acids. Its MS spectra showed a pseudomolecular ion [M-H]^−^ at *m/z* 137 and a prominent fragment ion at *m/z* 109 via loss of a CO group, corresponding to protocatechuic aldehyde. The UV spectra with λ_max_ at 280 and 310 nm were consistent with those of protocatechuic aldehyde reported in the literature^15^.

Three hydroxycinnamic acids, namely caffeic acid (peak 5), *p*-coumaric acid (peak 11) and ferulic acid (peak 12), were identified, giving the deprotonated molecule [M-H]^−^ at *m/z* 179, 163, and 193 and distinguished fragment ions at m/z 135, 119 and 135, respectively^15^. These hydroxycinnamic acids showed the loss of a CO_2_ group from the carboxylic acid functional group. Ferulic acid (peak 12) also lost a CH_3_ group demonstrating a [M-H-15]^−^ anion at m/z 178 (Table 1). The caffeic and ferulic acids in the samples were further identified by comparison with the HPLC Rt, UV and MS spectra of the authentic standards. Peak 15 had similar UV and MS spectra to ferulic acid, and was tentatively identified as ferulic acid isomer^16^.

Several derivatives of hydroxycinnamic acids were identified. Peaks 2 and 4 had deprotonated molecule [M-H]^−^ at *m/z* 253 and fragmentation patterns with ions at m/z 179 [caffeic acid –H]^−^ and 135 [caffeic acid –H-CO_2_]^−^, suggesting the possible occurrence of a caffeic acid residue. The UV λ_max_ of these two peaks were at 297 and 326 nm, which was the same as that of caffeic acid. The [M-H-74]^−^ ion at *m/z* 179 in peaks 2 and 4 is typical of that produced by the loss of a glycerol residue. Hence, peaks 2 and 4 were tentatively deduced as either 1-*O*-caffeoylglycerol or 2-*O*-caffeoylglycerol^17^, since it was not possible to ascertain the substituent group positions. Peak 8 had the same UV λ_max_ of 310 nm as that of *p*-coumaric acid. MS spectra showed a deprotonated molecule [M-H]^−^ at *m/z* 237 with fragment ions at *m/z* 163, 145 and 119. Fragment ions at *m/z* 163 and 119 appeared to correspond to deprotonated and decarboxylated *p*-coumaric acid, indicating the presence of *p*-coumaric acid. According to the above, this compound which lost the glycerol residue can produce a 163[M-H-74]^−^ anion. Therefore, this compound was tentatively identified as 1-*O-p*-coumaroylglycerol or 2-*O-p*-coumaroylglycerol^17^. The UV spectral characteristics of peaks 16 and 17 were similar to that of caffeic acid with λ_max_ at about 326 nm, suggesting derivatives of caffeic acid. They had deprotonated molecule [M-H]^−^ at *m/z* 415 and fragmentation patterns with ions at *m/z* 253, 179 [caffeic acid –H]^−^, 161 [caffeic acid –H-H_2_O]^−^ and 135 [caffeic acid –H-CO_2_]^−^, which supported the presence of a caffeic acid residue. The fragment ion of *m/z* at 253 (415-162) could correspond to dehydrated caffeic acid attached to the caffeoylglycerol. From a comparison of literature data of UV and MS spectra, these two peaks were tentatively identified as either 1,2-*O*-dicaffeoyglycerol or 1,3-*O*-dicaffeoyglycerol^17^. The deprotonated molecule ion [M-H]^−^ showed peaks of 20 and 21 at m/z 399 and 429, respectively. Both peaks contained the same ions at *m/z* 253, indicating that these two compounds may also contain a caffeoylglycerol residue. Peak 20 also showed two prominent ions at m/z 163 [*p*-coumaric acid-H]^−^ and 235 [M-H- *p*-coumaric acid]^−^, suggesting it could have been derived from the loss of a *p*-coumaric acid residue. For peak 21, some fragment ions at *m/z* 235 and 193 were detected. The ion at *m/z* 235 [M-H-194] indicated the loss of a ferulic acid moiety, while another ion at *m/z* 193, corresponded to deprotonated ferulic acid, suggested the presence of a ferulic acid moiety. After comparisons with the literature^17^, peaks 20 and 21 were tentatively identified as *p*-coumaroyl-caffeoylglycerol and feruloyl-caffeoylglycerol, respectively.

Flavanols. Peak 1 had a deprotonated molecular ion [M-H]^−^ at *m/z* 577 and fragment ions at *m/z* 425 and 289 (Table 1). The ion at *m/z* 425 [M-H-152] can be produced by the removal of a B-ring from the heterocyclic ring of catechin derivatives with the Retro-Diels-Alder rearrangement in the ESI negative ion mode^18^. The most intense ion at *m/z* 289 [M-H-289]^−^ was considered to be specific for a C—C interflavan linkage in the catechin dimer^19^. The UV spectra of peak 1 were similar to that of (+)-catechin with λ_max_ at 280 nm. Therefore, this peak was tentatively identified as procyanidin B, which was supported by its MS and UV spectra in the literature^20^.

**Figure 2:**
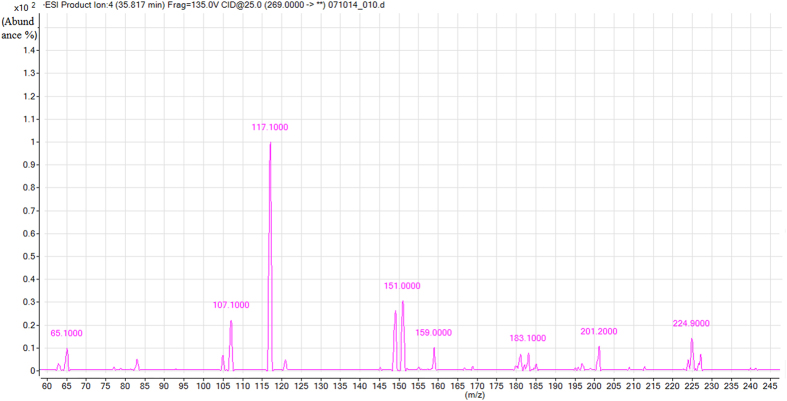
Mass spectra of apigenin of sorghum grains.

Flavones. Most flavones have two characteristic UV absorption bands in the region 250–285 nm and 320–385 nm. Band I(320–385 nm) is produced by the B-ring, while Band II(250–285 nm) by the A-ring^21^. Peaks 19 and 22 (Fig. 2) were positivity identified as the flavones luteolin and apigenin respectively by comparison with Rt, UV and mass spectral data (Table 1) of the authentic standards.

Flavanones and dihydroflavonols. Several flavanones were identified in sorghum grains. Peak 23 was positively identified as naringenin by comparing its Rt, UV and mass spectral data with the authentic standard. The deprotonated molecule ion [M-H]^−^ of peak 18 was at m/z 287, suggesting this compound might be eriodictyol or dihydrokaempferol with the molecular mass of 288. The peak 18 also showed the characteristic fragmentation ion at *m/z* 151 and UV spectra of eriodictyol, which exhibited a strong absorption in the region of 270–295 nm. Referring to MS and UV spectra of eriodictyol from the literature, peak 18 was tentatively identified as eriodictyol^15^. Peaks 6 and 10 showed the deprotonated molecule ions [M-H]^−^ at *m/z* 449 and 433, respectively. The fragmentation ions at *m/z* 287 and 151, corresponding to an eriodictyol moiety, were also observed. The UV spectra of these two peaks also showed similar absorption in the region 270–295 nm as that for eriodictyol, indicating they might be derivatives of eriodictyol. It is well known that most of the flavonoids can conjugate sugars, and their derivatives are present as the glycosides of *O-* or *C-* forms^22^. The peak 6 produced a fragmentation ion at *m/z* 287 [M-H-162]^−^ by possible loss of a glucoside, and the same fragmentation ion 287 [M-H-146]^−^ was also produced via the possible loss of a deoxyhexoside from peak 10. Therefore, peaks 6 and 10 were tentatively identified as eriodictyol-7-*O*-glucoside and eriodictyol deoxyhexoside, respectively^23^.

The compound of peak 13 was positivity identified as the hydroflavonol taxifolin by comparison of Rt, UV and mass spectral data (Table 1) with the authentic standard. Since peak 14 had similar UV and MS spectra to that of peak 13, it was tentatively identified as taxifolin isomer.

3-Deoxyanthocyanidins. Two compounds, corresponding to peaks 7 and 9, exhibited the distinctive UV spectra of 3-deoxyanthocyanidins. Typical 3-deoxyanthocyanidins produce two UV absorption peaks in the 450–560 nm region due to the B ring hydroxy cinnamoyl system and in the 240–280 nm region due to the A ring benzoyl system^21^. Compared with the Rt, MS and UV spectra of available standards, peaks 7 and 9 were unambiguously identified as luteolinidin and apigeninidin respectively.

### Profiles of phenolic compounds

The list of phenolic compounds found in the free and bound forms of the different sorghum genotypes under OT and HT are shown in Table 2. Nearly all phenolic compounds identified across the sample collection (Table 1) were also identified in each individual sorghum sample, except for the white sorghum PI563516, which contained fewer phenolic species. In addition, the range of phenolic compounds in the sorghum grains was larger in free form, and only brown sorghum IS 8525 showed a similar number of phenolic species in both for the free and bound fractions. The phenolic profile in AQL33/QL36, PI563516 and CHH2 genotypes was changed when grown under higher temperatures (Table 2). For example, the free forms of luteolin and apigenin were not detectable in CHH2 genotype under HT though present under OT (Fig. 1A,B).These results indicated that the phenolic profile in sorghum grains was affected strongly by genotype and also by growth temperature.

### Quantification of free, bound and total individual phenolic compounds

As total levels of phenolic compounds were presented in the previous paper^24^, individual phenolic compounds were evaluated. In order to more easily evaluate the results of the individual compounds, they were classified into five groups. Each compound was quantified in free and bound fractions and as total (free + bound).

Hydroxycinnamic acids. Three hydroxycinnamic acids were quantified in the sorghum genotypes: caffeic acid, ferulic acid and ferulic acid isomer (Table 3). Free, bound and total fractions of these compounds were significantly influenced when comparing treatments and genotypes (*p* ≤ 0.05). HT resulted in significantly lower free caffeic acid concentration than OT across the sorghum genotypes, except for CHH1, which showed higher levels at HT, but show no significant influences on free levels of ferulic acid and ferulic acid isomer of some genotypes (Ai4). HT showed no significant impacts on bound caffeic acid concentration of all genotypes, with the exception of CHH1 with higher levels at HT (*p* ≤ 0.05). The total levels of caffeic acid and ferulic acid varied widely (*p* ≤ 0.05) among these genotypes under both HT and OT, with PI563516 and IS 8525 having the lowest and highest levels, respectively (Table 3). The total ferulic acid concentration of all genotypes was extremely higher than other two phenolic acids, irrespective of temperature.

3-Deoxyanthocyanidins. Temperature, genotype and their interaction had significant influences on free, bound and total fractions of luteolinidin and apigeninidin accumulation (Table 4) (p ≤ 0.05). The accumulation trend of free luteolinidin showed a significant decline under HT in three genotypes, with the exception of two genotypes which did not contain the free luteolinidin, and free fraction of luteolinidin cannot be measured in PI 563516 at HT. The free form apigeninidin content changes followed the same pattern of free form luteolinidin under HT. Regardless of the treatment, Ai4 contained the highest free luteolinidin and apigeninidin concentrations under both temperatures. The levels of bound luteolinidin and apigeninidin showed various changes under HT. AQL33/QL36 and CHH2 accumulated the higher levels of bound luteolinidin and apigeninidin under both treatments, respectively.

Flavones. Two flavones: luteolin and apigenin were identified and quantified in the sorghum samples, and are shown in Table 4. Temperature, genotype and the interaction between the two factors had a significant effect on the flavone contents (*p* ≤ 0.05). Both free and bound luteolin and apigenin were not detected in the two sorghum genotypes of PI563516 and IS 8525. CHH2 contained detectable levels of free luteolin and apigenin at OT, but these flavones were not detected in this genotype at HT. Bound flavones accumulation also appears to be genotype dependent under HT. Generally, its concentration had no significant changes in CHH1, but significantly decreased in CHH2, AQL33/QL36 and Ai4 under HT. In fact, total luteolin and apigenin contents of CHH2 were higher than other genotypes under OT, but these of Ai4 reached to the highest level under HT.

Dihydroflavonol. The accumulation trend of two dihydroflavonols of taxifolin and taxifolin isomer are presented in Table 4 and their concentrations were significantly affected by temperature, genotype and their interaction (*p* ≤ 0.05). Compared to other sorghum genotypes, no flavonones in free nor bound form were detectable in PI563516 under both OT and HT. In general, free taxifolin and taxifolin isomer contents showed diverse changes in these genotypes under HT. However, the bound form of taxifolin was only detected in sorghum IS 8525 and was around 3 times higher than its free form in this genotype, but HT significantly reduced its concentration (*p* ≤ 0.05). Regardless of treatment, no bound taxifolin isomer was detected across all genotypes. These results suggest that these two compounds were mainly in the free form. The total values of taxifolin were generally higher in IS8525 as compared to others under both treatments.

Flavanones. Several flavanones (Table 1) were identified in the sorghum samples but only naringenin was quantified, due to a lack of available standards. Values of naringenin were significantly affected by genotype, temperature and their interaction (*p* ≤ 0.05). Apart from sorghum PI563516, all other sorghum genotypes contained naringenin in free and bound forms (Table 4). The bound naringenin level was significantly higher than free forms in five genotypes, irrespective of treatments. Total naringenin content in CHH2, Ai4 and IS 8525 was significantly lower at HT than OT, but significantly higher in AQL33/QL36 (*p* ≤ 0.05).

## Discussion

In this study several compounds were identified for the first time in sorghum grain. For example, feruloyl-caffeoylglycerol, which is known to be present in *Ananas comosus* L. leaves^17^, was tentatively identified in the sorghum grain. Similarly, two flavonoid compounds of eriodictyol deoxyhexoside and taxifolin isomer were also tentatively identified. It has been reported the presence of catechin in red sorghum PAN 3860^15^. This compound was not however detected in the six sorghum genotypes used in the present study. The two ubiquitous sorghum 3-deoxyanthocyanidins, apigeninidin (yellow) and luteolinidin (orange) were determined in our sorghum genotypes. The two other 3-deoxyanthocyanidins of 5-methoxyluteolinidin and 7-methoxyapigeninidin have not been detected in our samples, but been reported in sorghum genotypes with lemon-yellow pericarp previously^25^. Additionally, certain phenolic acids, such as gallic and cinnamic acids were identified in sorghum grain^26^, however these compounds were not detected in the present study. Some phenolic acid esters of glycerol, including dicaffeoylglycerol and caffeoylglycerol, have been found in other sorghum grains^15^, while the isomers of these phenolic acid esters of glycerol are reported for the first time in the present study. The results of the study confirm previous reports that the phenolic profile in sorghum grain is strongly affected by the genotype. There were some putative polyphenol peaks that were present in the HPLC-DAD-ESIMS chromatograms (Fig. 1) that could not be identified, likely due to their low UV signals in the DAD or unidentifiable molecular mass fragments.

The free, bound and total content of individual compounds in the sorghum genotypes in the present study was found to be in the range as previously reported^2^,^25^,^26^,^27^. Most of the phenolic compounds in sorghum grains were found in both free and bound forms (Fig. 1, Tables 3 and 4). For several individual compounds, such as caffeic acid, ferulic acid and naringenin, they mainly occurred in the bound form, which was in agreement with other studies^15^,^28^. It has been suggested that dietary intake of free or soluble conjugated forms rather than bound forms of phenolic compounds may have health benefits of protection against cardiovascular disease and certain types of cancer, since their gastrointestinal absorption is more rapid^24^,^29^. However, epidemiological studies also indicate that consuming whole grains with bound phenolic compounds could reduce the risk of colon cancer and other digestive cancers. This may be related to the bound phenolic compounds surviving stomach and intestinal digestion and then being degraded by the microflora and absorbed in the colon to prevent colon cancer^30^.

The profiles and levels of phenolic compounds cannot be simply attributed to the sorghum grain colour. It has been reported that five different red sorghum genotypes were planted under the same growth condition, but some individual phenolics, such as luteolin, were only detected in red 99LGWO50^2^. In the present study, luteolin was absent in red sorghum AQL33/QL36 in comparison with other red genotypes. White sorghum PI563516 contained the least amount of flavonoids, with some common flavonoids, such as luteolin, apigenin and naringenin, being absent in this genotype (Table 2). In additon, luteolinidin and apigeninidin were present in white sorghum 02CA4796^9^, but the levels were lower than in PI563516 in the present study. It has been reported that the biosynthesis of phenolic compounds in plants is regulated by enzymes such as phenylalanine ammonia-lyase (PAL), chalcone isomerase (CHI) and UDP glucose flavonoid-glucosyltransferase (UFGT)^31^. Enzyme activities, which have potential influences on phenolic biosynthesis, varied among genotypes. Mccallum and Walker^32^ reported that red wheat genotypes contained higher levels of both PAL and CHI than white genotypes during early grain development, which can lead to higher synthesis of flavonoids. The similar results were also observed in two different apple genotypes, and PAL, CHI and UFGT activities of red-skinned genotype was several-fold higher than green-skinned genotype at all stages, so a greater accumulation of flavonoids in red-skinned genotype^33^. In the present study, similar results were obtained in sorghum grains as well. The total 3-deoxyanthocyanidins, flavones, dihydroflavonol and flavanone concentrations of colored sorghum were higher than white sorghum, indicating that these PAL, CHI and UFGT enzyme activities might be higher in colored sorghum.

The impact of high temperature on sorghum grain phenolic compounds was diverse across sorghum genotypes. The possible reason was that the phenolic metabolism pathway was also influenced by temperature. As the first step in the biosynthesis of phenolic compounds was catalyzed by PAL, the PAL activity might have correlation with phenolic levels^32^. Rivero *et al*.^34^ reported that high temperature could increase the PAL activity in tomato, and the total phenolic compounds were increased under high temperature stress. Based on the present study, it can be proposed that the phenolic changes under high temperature in sorghum grain may be caused by PAL activity change. For individual polyphenols, their changes might be related to some specific enzyme activity changes. For example, anthocyanin concentration of grape berries grown at a constant 30 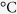 was lower than those grown at 30 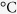/15 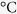
(day/night), and anthocyanin accumulation correlated strongly with UFGT activity^35^. However, the biosynthetic pathways of 3-deoxyanthocyanidin in sorghum may be different from anthocyanin biosynthesis in other plants, and the enzymes involved in 3-deoxyanthocyanidin synthesis have still remained unexplored^36^. In the present research, it was noted that the abundance of free luteolinidin and apigeninidin was markedly decreased under high temperature, and these changes might be related to enzymes. Further research is required to explore enzymes in the metabolic pathway of 3-deoxyanthocyanidin of sorghum grains under high temperature conditions.

The antioxidant activity of total phenolic compounds of sorghum grain determined by 2′–azinobis (3-ethylbenzothiazoline-6-sulfonic acid) diammonium salt (ABTS) and 2-2-diphenyl-1-picrylhydrazyl (DPPH) assays were presented in the previous paper^24^, and their antioxidant activity was contributed by profiles and levels of individual polyphenols. Chandrasekara and Shahidi^37^ reported that two millet grain genotypes: Proso and Pearl with less complex profiles of phenolics showed the lowest antioxidant activity. Similar changes in sorghum grain were also presented in the present experiment. PI563516 had lower antioxidant activity than others^24^, and the profile of phenolics was the least complex (Table 2). For individual phenolic acids, total antioxidant activity of ferulic acid is higher than caffeic acid. Additionally, not all individual flavonoids show high antioxidant activity. For example, apigenidin, luteolin and taxifolin have higher antioxidant activity than naringenin and apigenin^21^. Therefore, the levels of individual phenolic compounds might decide the antioxidant activity of sorghum grain. Durum wheat pasta with different levels of red sorghum flour or white sorghum flour was made, and it was found that the wheat pasta with red sorghum flour which contained high levels of individual phenolic acids showed higher antioxidant capacity than white sorghum flour^11^. In this research, IS 8525, CHH1 and CHH2 had higher levels of ferulic acid, caffeic acid than others, and higher antioxidant activity was obtained in these genotypes as well^24^. As the antioxidant activity of sorghum grain had a strong correlation with phenolic content, HT can also change the antioxidant activity of sorghum grain. Individual polyphenols of IS8525 significantly declined under HT, and total antioxidant activity of it decreased^24^. Compared to other research, the individual phenolics of strawberry increased when growth temperature increased from 18/12 °C to 30/22 °C (day/night), resulting in a significant increase in antioxidant capacity^13^. The phenolic changes of sorghum grain under temperature treatments are important for humans, due to their free radical scavenging and biological properties. In this research, the sorghum genotype IS 8525 had extremely high levels of ferulic acid, caffeic acid and taxifolin compared to others under both temperatures, suggesting that this genotype may be suitable for use in health-functional food formulation.

In conclusion, with the use of the rapid analytical procedure of HPLC-DAD-ESIMS, the phenolic compounds in 6 sorghum genotypes under two different growth temperatures were determined. The profiles and contents of phenolic compounds in sorghum grains were significantly influenced by the genotype, growth temperature and their interaction. This study has attempted to further our understanding of the variations of polyphenols in sorghum under the projected higher temperature conditions in certain regions, as well as indicating the potential for particular sorghums to be used as a nutritious food source depending on growth temperature and genotype.

## Methods

### Sorghum genotypes

**Figure 3:**
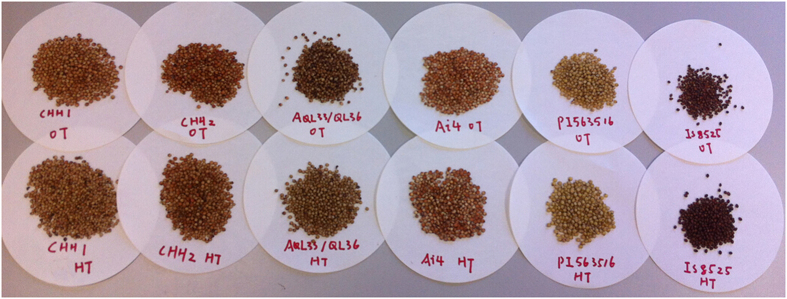
Six genotypes of mature sorghum grain under OT and HT growth conditions.

Six sorghum genotypes including three red pericarp hybrid lines (CCH1, CCH2 and AQL33/QL36) and three inbred lines (Ai4 red pericarp; PI563516, white pericarp; IS 8525, brown pericarp) were used in this study (Fig. 3).

### Experimental design

The details of sorghum growing conditions have been described previously^38^. Briefly, two separate growth chambers were used to control all environmental factors and provide two temperature regimes: optimal temperature (OT, 32 °C day/21 °C night) and high temperature (HT, 38 °C day/21 °C night) at the Controlled Environment Facility, Queensland Bioscience Precinct, The University of Queensland, Brisbane, Australia, 2012. The night temperature was gradually increased at a rate of 3 °C/hour to the maximum temperatures commencing from 1 h after the light period began. The maximum temperatures were kept constant for 7 h and 3 h in the OT and HT chambers, respectively. The temperature was then decreased at a rate of 3 °C/hour until the minimum temperature was obtained in both chambers^38^. Therefore, the main environmental difference between the two treatments was only the temperature in the middle of the light period. The experiment was a completely randomised block design (using 6 genotypes) within two temperature treatments and three replications (individual plants). Grains were harvested at maturity, manually cleaned and air-dried until a moisture content of around 10% was reached. The dried samples from each replicate were individually vacuum-packed in moisture proof packaging and kept at −20 °C in the dark until analysis.

### Extraction of free and bound phenolic compounds

The whole grain samples were milled (CEMOTEC 1090, Foss Tecator, Hoganäs, Sweden) to pass 100% through a 500 μm sieve. The free and bound phenolic compounds were extracted^15^. All extractions were evaporated to dryness using a rotary vacuum evaporator. The resulting solid was re-dissolved in methanol to a final volume of 10 ml and stored under N_2_ at −20 °C in the dark until analysis.

### HPLC-DAD-ESIMS analyses

An Agilent 1290 UHPLC system equipped with binary pump, autosampler, thermostated column compartment, degasser, and diode array detector (DAD) was coupled to an Agilent 6460 LC-QQQ LC-MS/MS system (Agilent Technologies, Palo Alto, CA, USA). The separation of phenolic compounds was performed on a Kinetex XB-C 18 reversed phase-HPLC column (5 μm, 250 × 4.6 mm, Phenomenex, Torrance, CA, USA). The DAD was set to scan between 190 and 600 nm at steps of 2 nm. Solvent A consisted of 0.1% formic acid in LC-MS grade water (Honeywell Burdick & Jackson, Gillman, SA, Australia), and solvent B was LC-MS grade acetonitrile (Honeywell Burdick & Jackson, Gillman, SA, Australia). The sample injection volume was 5 μl with the following linear gradient elution: 5%–15% B (5 min), 15%–50% B (40 min), 50%–70% B (2 min), 70%–100% B (1 min), 100% B (7 min), 100%-5% B (1 min), 5% B (9 min). The flow rate was 0.5 ml/min.

Mass spectra were performed in the ESI negative mode with a scan time of 2000 MS under the following conditions: gas (N2) 5 L/min at 300 °C, nebulizer 45 psi, sheath gas (N_2_) 11 L/min at 250 °C, capillary voltage −3.5 kV and nozzle voltage −500 V. Phenolic compounds were detected by full scan ranging from *m/z* 50 to 1300.

### Identification and quantification of polyphenols

The phenolic compounds in sorghum samples were identified by their UV-Vis and ESIMS spectra. The authentic standards were also used to identify phenolic compounds based on the chromatographic comparisons. The external standard method was used to quantify phenolic compounds in samples under the above HPLC-DAD conditions. Standards were dissolved in methanol (1 mg/ml each), and diluted to several concentrations ranging from 0.001 to 0.1 mg/ml. Data acquisition, peak integration and calibrations were performed with the Agilent ChemStation software. Peak areas compared with calibration curves of the respective standards were used to calculate levels of phenolic compounds, and results were expressed as μg/g sample (dry basis, db). Quantification of taxifolin isomer and ferulic acid isomer were calculated using the calibration curve for taxifolin and ferulic acid, respectively.

### Statistical analysis

All data were reported as means ± SD of triplicate independent experiments analysed in triplicate. The main effects of genotype and temperature and their interaction were investigated by two-way or one way ANOVA with SPSS Statistics V20 (IBM Corp., Armonk, NY, USA). Significant differences were considered when *p* ≤ 0.05.

## Additional Information

**How to cite this article**: Wu, G. *et al*. Growth temperature and genotype both play important roles in sorghum grain phenolic composition. *Sci. Rep.* **6**, 21835; doi: 10.1038/srep21835 (2016).

## Figures and Tables

**Table 1:** Identification of free and bound phenolic compounds in sorghum grains by HPLC-DAD-ESIMS and respective standards (std).

Peak No.	Rt^a^ (min)	λ_max_ (nm)	*m/z* [M - H]^−^	*m/z* MS^2^ (Abundance %)	Tentative identification
1	10.9	280	577	425 (60), 289 (26)	Procyanidin B1
2	13.1	298sh^b^, 326sh	253	179 (1), 161 (78), 135 (55)	2-*O*-caffeoylglycerol
3	13.5	280. 310	137	109 (15)	Protocatechuic aldehyde
4	14.4	298sh, 326sh	253	179 (1), 161 (78), 135 (55)	1-*O*-caffeoylglycerol
5	15.0	297, 322	179	135 (100)	Caffeic acid (std)
6	15.8	282	449	287 (8), 151 (100), 135 (41)	Eriodictyol-7-*O*-glucoside
7	16.1	280, 490	269	241 (41), 225 (27), 169 (19), 133 (30)	Luteolinidin (std)
8	17.7	310	237	163 (20), 145 (50), 119 (31)	2-*O*-*p-*coumaroylglycerol
9	18.2	275, 470	253	225 (10), 209 (70), 179 (40), 117 (65)	Apigeninidin (std)
10	19.1	283	433	287 (100), 151 (59)	Eriodictyol deoxyhexoside
11	19.2	310	163	119 (100)	*p-*coumaric acid
12	20.8	295, 325	193	178 (42), 134 (100)	Ferulic acid (std)
13	21.6	288	303	285 (100), 217 (9), 177 (18), 125 (35)	Taxifolin (std)
14	24.3	285	303	285 (10), 177 (18), 125 (35)	Taxifolin isomer
15	26.7	295, 325	193	134 (100)	Ferulic acid isomer
16	28.5	300sh, 326sh	415	253 (100), 179 (100) , 161 (11), 135 (85)	1,2-*O*-dicaffeoylglycerol
17	29.4	300sh, 326sh	415	253 (100), 179 (100) , 161 (11), 135 (85) 135(85),	1,3-*O*-dicaffeoylglycerol
18	30.6	287	287	151(10)	Eriodictyol
19	31.1	252, 347	285	241 (1), 217 (3), 199 (3), 175 (3), 151 (17), 133 (13), 107 (3)	Luteolin (std)
20	33.4	219, 315	399	253 (80), 235 (11), 179 (25), 163 (86), 145 (35), 119 (100),135 (57)	Coumaroyl-caffeoylglycerol
21	34.2	295sh, 325sh	429	253 (70), 235 (11), 193 (100), 175 (32), 161 (53), 135 (41)	Feruloyl-caffeoylglycerol
22	35.9	266, 322	269	225 (10), 201 (30), 183 (10), 149 (32), 117 (100)	Apigenin (std)
23	36.2	295	271	177 (10), 151 (50), 119 (20), 107 (20)	Naringenin (std)

^a^Rt = Retention time.

^b^sh = shoulder.

**Table 2:** Profiles of phenolic compounds in the six genotypes of sorghum grain grown at OT and HT.

Genotype	Peak number^a^			
	OT		HT	
	Free	Bound	Free	Bound
CCH1	1, 2, 3, 4, 5, 6, 7, 8, 10, 12, 13, 15, 16, 17, 19, 20, 21, 23	5, 7, 9, 11, 12, 18, 19, 20, 23	1, 2, 3, 4, 5, 6, 7, 8, 10, 12, 13, 15, 16, 17, 19, 20, 21, 23	5, 7, 9, 11, 12, 18, 19, 20, 23
CCH2	1, 2, 3, 4, 5, 7, 8, 10, 12, 13, 14, 15, 17, 19, 20, 21, 22, 23	5, 7, 9 11, 12, 15, 19, 20, 22, 23	1, 2, 3, 4, 5, 7, 8, 10, 12, 13, 14, 15, 17, 20, 21, 23	5, 7, 9 11, 12, 15, 19, 20, 22, 23
AQL33/QL36	1, 2, 3, 4, 5, 6, 8, 9, 10, 12, 13, 15, 16, 17, 20, 21, 22, 23	5, 7, 9, 11, 12, 17, 18, 19, 20, 22, 23	1, 2, 3, 4, 5, 6, 8, 9, 10, 12, 13, 15, 16, 17, 20, 21, 23	5, 7, 9, 11, 12, 17, 18, 19, 20, 22, 23
Ai4	1, 2, 4, 5, 7, 8, 9, 12, 13, 14, 15, 17, 19, 20, 21, 22, 23	5, 7, 9, 11, 12, 18, 19, 20, 22, 23	1, 2, 4, 5, 7, 8, 9, 12, 13, 14, 15, 17, 19, 20, 21, 22, 23	5, 7, 9, 11, 12, 18, 19, 20, 22, 23
PI563516	1, 2, 4, 5, 7, 8, 9, 12, 16, 17, 20, 21	5, 11, 12, 18	1, 2, 4, 5, 8, 12, 16, 17, 20, 21	5, 11, 12, 18
IS 8525	1, 2, 3, 4, 5, 6, 9, 10, 12, 13, 14, 15, 16, 17, 20, 21, 23	2, 3, 4, 5, 7, 8, 9, 11, 12, 13, 15, 16, 18, 20, 23	1, 2, 3, 4, 5, 6, 9, 10, 12, 13, 14, 15, 16, 17, 20, 21, 23	2, 3, 4, 5, 7, 8, 9, 11, 12, 13, 15, 16, 18, 20, 23

^a^Peak number identified in Table 1.

**Table 3:** Free, bound and total hydroxycinnamic acid content (μg/g, dry base (db)) of six genotypes of sorghum grain grown at OT and HT.

			Genotypes					
Hydroxycinnamic acids			CHH1	CHH2	AQL33/QL36	Ai4	PI563516	IS 8525
CA	Free	OT	5.98 ± 0.23 aA	7.19 ± 0.54 bB	7.86 ± 0.49 bB	13.12 ± 0.74 cB	6.54 ± 0.32 aB	33.72 ± 4.22 dB
		HT	7.63 ± 0.50 cB	4.45 ± 0.66 aA	6.17 ± 0.49 bA	9.98 ± 0.65 dA	4.22 ± 0.97 aA	18.84 ± 1.46 eA
	Bound	OT	12.14 ± 1.05 cA	12.92 ± 0.76 cA	9.37 ± 0.35 bA	5.56 ± 0.47 aA	4.80 ± 0.22 aA	13.89 ± 0.34 cA
		HT	15.89 ± 0.81 dB	11.38 ± 1.87 cA	9.54 ± 0.03 bA	6.88 ± 0.17 aA	5.01 ± 1.12 aA	14.18 ± 0.15 dA
	Total	OT	18.12 ± 1.27 bA	20.10 ± 0.21 cB	17.22 ± 0.83 bB	18.67 ± 0.27 bB	11.34 ± 0.54 aB	47.61 ± 3.89 dB
		HT	23.51 ± 0.31 cB	15.83 ± 2.53 bA	15.71 ± 0.52 bA	16.68 ± 0.82 bA	9.23 ± 0.97 aA	33.02 ± 1.61 dA
FA	Free	OT	11.06 ± 1.16 cA	33.16 ± 0.35 eB	0.63 ± 0.06 aA	1.06 ± 0.13 bA	0.53 ± 0.11 aA	26.82 ± 2.94 dB
		HT	10.21 ± 0.17 cA	26.00 ± 3.35 eA	1.11 ± 0.11 bB	1.18 ± 0.01 bA	0.61 ± 0.12 aA	16.74 ± 5.17 dA
	Bound	OT	71.07 ± 1.77 bA	86.09 ± 0.40 cB	35.43 ± 0.97 aA	35.85 ± 0.33 aA	34.88 ± 1.75 aA	125.50 ± 1.96 dB
		HT	73.18 ± 0.81 cA	69.02 ± 0.24 cA	45.22 ± 0.16 bB	40.45 ± 1.62 aB	37.17 ± 0.51 aB	116.13 ± 5.30 dA
	Total	OT	82.13 ± 0.61 bA	119.25 ± 0.05 cB	36.05 ± 0.91 aA	36.91 ± 0.45 aA	35.41 ± 1.84 aA	152.32 ± 7.06 dB
		HT	83.39 ± 0.64 cA	95.02 ± 3.11 dA	46.32 ± 0.06 bB	41.62 ± 1.61 aB	37.78 ± 0.46 aB	132.88 ± 10.48^eA^
FAI	Free	OT	1.76 ± 0.04 aA	7.62 ± 0.44 cB	3.40 ± 0.16 bA	1.65 ± 0.14 aA	nd	3.60 ± 0.20 bB
		HT	2.74 ± 0.13 bB	4.48 ± 0.97 cA	4.17 ± 0.48 cA	1.38 ± 0.07 aA	nd	1.79 ± 0.38 aA
	Bound	OT	nd	0.24 ± 0.01 aB	nd	nd	nd	5.34 ± 0.25 bB
		HT	nd	0.14 ± 0.02 aA	nd	nd	nd	4.19 ± 0.35 bA
	Total	OT	1.76 ± 0.04 aA	7.86 ± 0.45 cB	3.40 ± 0.16 bA	1.65 ± 0.14 aB	na	9.00 ± 0.63 dB
		HT	2.74 ± 0.13 bB	4.62 ± 0.97 cA	4.17 ± 0.48 cB	1.38 ± 0.07 aA	na	5.98 ± 0.73 dA

^a,b,c,d,e^Values with different superscripts in the same row are significantly different (*p* ≤ 0.05).

^A,B^Values with different superscripts in the same column in the same dependent variable are significantly different (*p* ≤ 0.05).

Abbreviations: nd = not detected; na = data not available; CA = caffeic acid; FA = ferulic acid; FAI = ferulic acid isomer.

**Table 4:** Free, bound and total individual flavonoids content (μg/g, db) of six genotypes of sorghum grain grown at OT and HT.

			Genotypes					
Flavonoids			CHH1	CHH2	AQL33/QL36	Ai4	PI563516	IS 8525
3-Deoxyanthocyanidin								
LUT	Free	OT	0.65 ± 0.04 aB	2.33 ± 0.13 bB	nd	14.24 ± 0.98 dB	4.40 ± 0.34 c	nd
		HT	0.13 ± 0.02 aA	0.36 ± 0.01 bA	nd	9.22 ± 0.18 cA	nd	nd
	Bound	OT	1.92 ± 0.55 bA	3.43 ± 0.06 cB	3.88 ± 0.23 cA	0.69 ± 0.07 aA	nd	1.62 ± 0.25 bA
		HT	3.47 ± 0.28 cB	1.96 ± 0.21 bA	4.10 ± 0.48 dA	1.37 ± 0.13 aB	nd	1.58 ± 0.51 abA
	Total	OT	2.57 ± 0.51 bA	5.76 ± 0.08 eB	3.88 ± 0.23 cA	14.93 ± 1.05 fB	4.40 ± 0.34 dB	1.62 ± 0.25 aA
		HT	3.60 ± 0.30 cB	2.31 ± 0.21 bA	4.10 ± 0.48 cA	10.59 ± 0.31 dA	na	1.58 ± 0.51 aA
API	Free	OT	2.74 ± 0.21 bB	2.49 ± 0.01 abB	2.25 ± 0.18 aB	23.24 ± 1.29 cB	2.48 ± 0.21 ab	2.05 ± 0.08 aB
		HT	0.38 ± 0.02 aA	0.64 ± 0.08 bA	0.33 ± 0.01 aA	14.52 ± 0.71 cA	nd	0.60 ± 0.02 bA
	Bound	OT	4.66 ± 0.14 bA	23.84 ± 1.73 dB	12.16 ± 1.32 cA	4.70 ± 0.16 bA	nd	2.27 ± 0.18 aA
		HT	7.79 ± 0.08 cB	16.88 ± 3.79 eA	11.57 ± 1.10 dA	6.20 ± 0.31 bB	nd	2.43 ± 0.65 aA
	Total	OT	7.40 ± 0.06 cA	26.33 ± 1.71 eB	14.41 ± 1.51 dB	27.93 ± 1.46 eB	2.48 ± 0.21 a	4.33 ± 0.26 bB
		HT	8.16 ± 0.06 bB	17.52 ± 3.71 dA	11.89 ± 1.09 cA	20.72 ± 0.40 eA	na	3.03 ± 0.63 aA
Flavones								
LU	Free	OT	0.92 ± 0.17 aA	10.91 ± 0.57 c	nd	4.04 ± 0.07 bA	nd	nd
		HT	0.84 ± 0.10 aA	nd	nd	5.57 ± 0.35 bB	nd	nd
	Bound	OT	3.70 ± 0.03 bA	5.05 ± 0.74 cB	0.73 ± 0.06 aB	0.91 ± 0.02 aB	nd	nd
		HT	3.92 ± 0.12 bA	3.62 ± 0.13 bA	0.54 ± 0.03 aA	0.52 ± 0.03 aA	nd	nd
	Total	OT	4.62 ± 0.14 bA	15.96 ± 1.30 cB	0.73 ± 0.06 aB	4.95 ± 0.09 bA	na	na
		HT	4.76 ± 0.22 cA	3.62 ± 0.13 bA	0.54 ± 0.03 aA	6.09 ± 0.37 dB	na	na
AP	Free	OT	nd	1.42 ± 0.00 b	0.36 ± 0.08 a	1.45 ± 0.13 bA	nd	nd
		HT	nd	nd	nd	2.09 ± 0.37 B	nd	nd
	Bound	OT	nd	2.61 ± 0.13 cB	1.80 ± 0.21 bB	0.86 ± 0.02 aA	nd	nd
		HT	nd	1.20 ± 0.17 abA	1.27 ± 0.04 bA	1.16 ± 0.02 aB	nd	nd
	Total	OT	na	4.03 ± 0.13 bB	2.16 ± 0.30 aB	2.30 ± 0.11 aA	na	na
		HT	na	1.20 ± 0.17 aA	1.27 ± 0.04 aA	3.24 ± 0.40 bB	na	na
Dihydroflavonol								
TA	Free	OT	1.78 ± 0.15 bA	7.92 ± 0.82 dB	2.39 ± 0.05 cB	1.21 ± 0.01 aA	nd	12.85 ± 0.78 eA
		HT	2.35 ± 0.11 cB	4.15 ± 0.21 dA	1.85 ± 0.10 bA	1.55 ± 0.01 aA	nd	12.36 ± 2.38 eA
	Bound	OT	nd	nd	nd	nd	nd	41.33 ± 1.10 B
		HT	nd	nd	nd	nd	nd	31.91 ± 0.03 A
	Total	OT	1.78 ± 0.15 bA	7.92 ± 0.82 dB	2.39 ± 0.05 cB	1.21 ± 0.01 aA	nd	54.18 ± 1.88 eB
		HT	2.35 ± 0.11 cB	4.15 ± 0.21 dA	1.85 ± 0.10 bA	1.55 ± 0.01 aA	nd	44.27 ± 2.34 eA
TAI	Free	OT	0.86 ± 0.07 aA	12.97 ± 0.54 cB	nd	11.21 ± 0.08 cB	nd	3.60 ± 0.20 bB
		HT	1.55 ± 0.04 aB	5.45 ± 0.48 bA	nd	9.41 ± 0.28 cA	nd	1.66 ± 0.50 aA
	Bound	OT	nd	nd	nd	nd	nd	nd
		HT	nd	nd	nd	nd	nd	nd
	Total	OT	0.86 ± 0.07 aA	12.97 ± 0.54 cB	na	11.21 ± 0.08 cB	na	3.60 ± 0.20 bB
		HT	1.55 ± 0.04 aB	5.45 ± 0.48 bA	na	9.41 ± 0.28 cA	na	1.66 ± 0.50 aA
Flavanone								
NAR	Free	OT	1.37 ± 0.53 bA	2.03 ± 0.16 cB	0.62 ± 0.08 aA	1.63 ± 0.04 bcA	nd	1.97 ± 0.10 cB
		HT	1.54 ± 0.21 dA	4.15 ± 0.21 dA	0.64 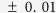 aA	1.47 ± 0.06 dA	nd	1.25 ± 0.03 cA
	Bound	OT	9.40 ± 1.41 cA	6.46 ± 0.25 bB	4.28 ± 0.44 aA	20.58 ± 0.75 dB	nd	10.34 ± 0.93 cB
		HT	9.32 ± 0.91 cdA	4.87 ± 0.09 aA	6.45 ± 0.83 bB	10.54 ± 0.59 dA	nd	8.80 ± 0.38 cA
	Total	OT	10.76 ± 1.94 cA	8.48 ± 0.08 bB	4.90 ± 0.35 aA	22.21 ± 0.78 eA	nd	12.31 ± 0.83 dB
		HT	10.86 ± 1.12 cA	5.75 ± 0.04 aA	7.09 ± 0.84 bB	12.01 ± 0.53 dB	nd	10.05 ± 0.42 cA

^a,b,c,d,e^Values with different superscripts in the same row are significantly different (*p* ≤ 0.05).

^A,B^Values with different superscripts in the same column in the same dependent variable are significantly different (*p* ≤ 0.05).

Abbreviations: nd = not detected; na = data not available; LUT = luteolinidin; API = apigeninidin; LU = luteolin; AP = Apigenin; TA = taxifolin; TAI = taxifolin isomer; NAR = naringenin.
